# Risk for Cardiovascular Death Associated With Waist Circumference and Diabetes: A 9-Year Prospective Study in the Wan Shou Lu Cohort

**DOI:** 10.3389/fcvm.2022.856517

**Published:** 2022-04-26

**Authors:** Man Li, Ping Zhu, Shu-xia Wang

**Affiliations:** Department of Geriatrics, The Second Medical Center and National Clinical Research Center for Geriatric Diseases, Chinese People's Liberation Army General Hospital, Beijing, China

**Keywords:** waist circumference, diabetes, obesity, cardiovascular death, cardiovascular-outcome

## Abstract

**Background:**

It has been reported that obesity and diabetes are both risk factors for the development of cardiovascular diseases (CVDs). However, recent articles reported that compared with body mass index, waist circumference (WC) can better reflect obesity, more closely related to visceral fat tissue which is positively associated with an increased risk of cardiovascular death. Moreover, few studies have investigated the prognostic value of both WC and diabetes during a long-term follow-up. We aimed to investigate whether the higher level of WC measurements and diabetes were able to predict cardiovascular mortality in the general population.

**Methods:**

In this prospective cohort study, a total of 1,521 consecutive subjects free of clinical CVD were included. The endpoint was cardiovascular death. The Kaplan–Meier method and Cox regression models were used to evaluate the cumulative risk of the outcome at different WC levels with or without diabetes.

**Results:**

During a median follow-up of 9.2 years, 265 patients died due to cardiovascular conditions. Kaplan–Meier survival estimates indicated that the patients with higher levels of WC (WC > 94 cm) coexisted with diabetes had a significantly increased risk of cardiovascular death (log-rank *p* < 0.05). After adjustment for potential confounders, multiple COX regression models showed that the incidence of cardiovascular death was significantly higher when patients with high WC coexisted with diabetes mellitus (hazard ratio: 3.78; 95% CI: 3.35–3.98; *p* < 0.001).

**Conclusion:**

Patients with high WC and diabetes represent a high-risk population for cardiovascular death. WC and diabetes may provide incremental prognostic value beyond traditional risks factors.

## Background

Obesity has become a major health problem worldwide. A high amount of adiposity is associated with an increased risk of various diseases, including cardiovascular disease (CVD), diabetes, and cancer. It is recommended that obesity, which is usually defined as excessive body fat and damage to health, can no longer be assessed only by body mass index (BMI) (1). In epidemiological studies, BMI is often used to define overweight and obesity. However, BMI has a low sensitivity and there are large individual differences between body fat percentages, partly due to age, gender, and race (2). Recent studies have indicated that visceral adipose tissue (VAT) was a major threat to cardiovascular risk (3). It has been reported that compared with BMI, waist circumference (WC) can better reflect body fat distribution, more closely related to VAT (4). In particular, abdominal obesity is a better predictor of cardiovascular events than BMI (5). Higher cardiometabolic risk is also related to the location of excess fat in VAT and ectopic reservoirs (such as muscle and liver), when the ratio of fat to lean body mass increases (for example, normal body weight for metabolic obesity) (6).

Moreover, previous studies have demonstrated that compared with BMI-matched non-type 2 diabetic patients, type 2 diabetic patients have a larger WC and more VAT than BMI-matched individuals without type 2 diabetes mellitus (DM) (7). These patients are all susceptible to cardiovascular abnormalities and CVDs; their coexistence should further increase the risk of cardiovascular outcomes (8). These data indicate that obese patients with diabetes may be more common than large epidemiological studies have shown and require more urgent attention. Relying solely on BMI to assess its prevalence may hinder future interventions for obesity prevention and control. Moreover, long-term research on WC and diabetes-related to predictive value is still very limited (9). Therefore, this study ought to investigate the prognostic value of WC on cardiovascular death during a long-term follow-up.

## Methods

### The Study Population

**Figure 1:**
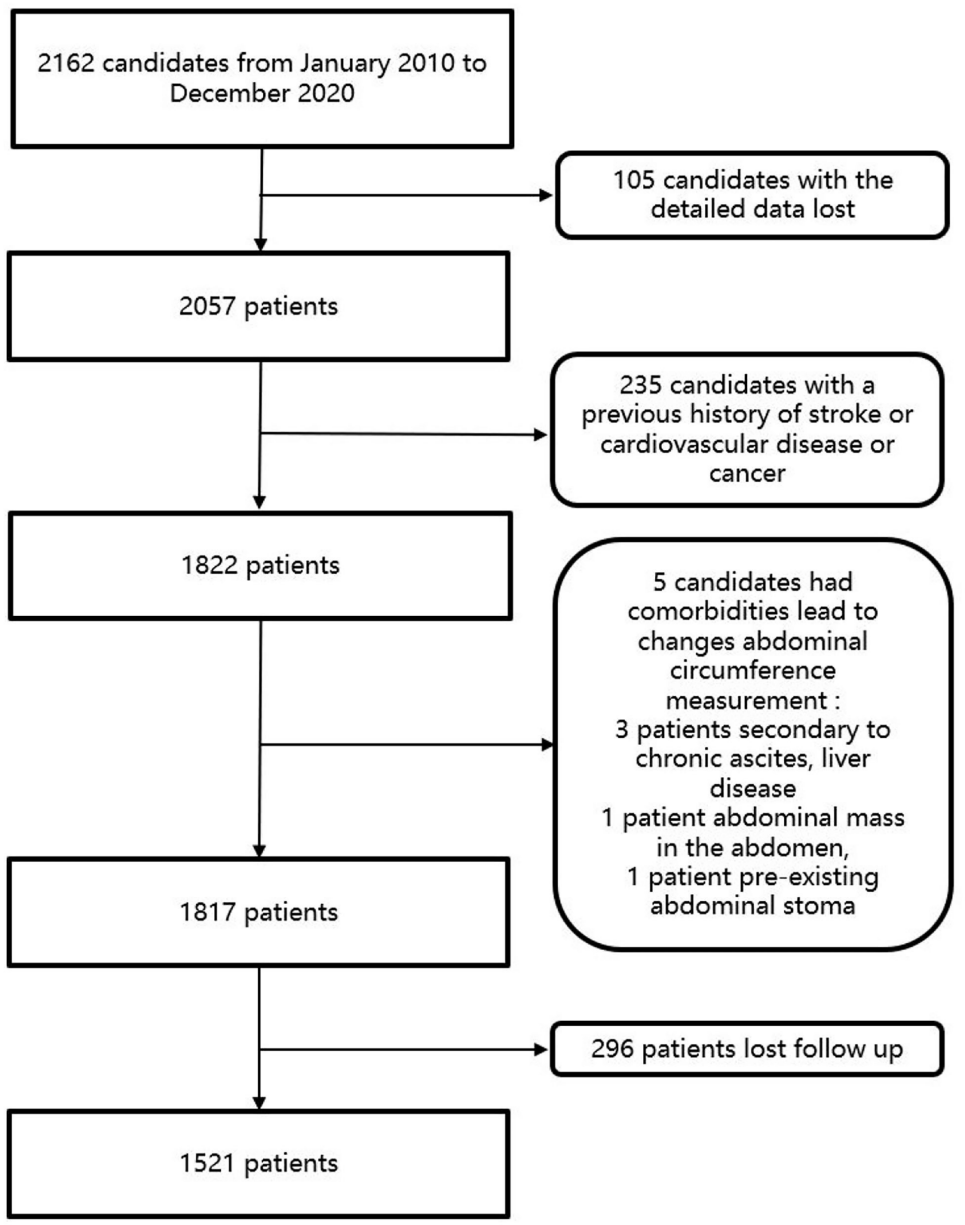
The flowchart of the study.

From January 2010 to December 2020, a total of 2,162 people in Wan Shou Lu Street were included in the study. Among these participants, patients who had a history of stroke, CVD, or cancer were excluded from the study. In this study, patients were excluded if the patients could not give informed consent, had comorbidities leading to changes in abdominal circumference measurement (secondary to chronic ascites, liver disease, cancer, intestinal obstruction, abdominal mass in the abdomen, pre-existing abdominal stoma, and incisional hernia). Patients who were pregnant were also excluded from the study. Participants were also excluded if their blood samples or detailed data were not available. Candidates who lost follow-up were also excluded from the study. Finally, a total of 1,521 patients were included in this study. The flowchart of the study was listed in Figure 1.

### Baseline Measurement

The information on sociodemographic characteristics, medical history, and lifestyle factors was accessed by a group of trained and experienced personnel researchers through a face-to-face interview using a detailed questionnaire. A structured questionnaire was used to collect information on patients. The questionnaire was designed by a group of experts and was previously piloted in a subsample of participants. The information on participants gathered in the questionnaire was reviewed by two investigators and encoded to protect the confidentiality of patients. Anthropometric measures (height, weight, and WC) were measured in triplicate by registered nurses using standardized techniques, calibrated scales, and wall-mounted stadiometers, respectively. WC was measured in duplicate by trained staff, halfway between the last rib and the iliac crest using an anthropometric tape parallel with the floor. Blood pressure was measured in triplicate using a validated semiautomatic oscillometer (OMRON^®^ M4-1), OMRON, automation (China) co., LTD. Measurement averages were calculated later for analysis purposes. The measures of the participants were collected at the time of registration. Anthropometric measurements include weight in kilograms, height in meters, hips circumference, and WC. WC was measured on the mid-axillary line between the lowest border of the thoracic cage and the top of the iliac crest to the nearest 0.1 cm. DM is defined as the presence of diabetic symptoms and resting plasma glucose concentration ≥200 mg/dl, fasting plasma glucose concentration ≥126 mg/dl, or 2-h plasma glucose concentration ≥200 mg/dl, or the use of an oral hypoglycemic agent, or insulin at the time of admission. BMI is calculated as weight/height^2^. After resting for 5 min, the right arm is used in a sitting position and standard or automated equipment is used to measure blood pressure, and the average of the two blood pressure is used as measurements as the final blood pressure. The patient was considered to have hypertension with BP>140/90 mmHg or received antihypertensive medication. Hyperlipidemia is defined as known but untreated dyslipidemia or current treatment with lipid-lowering drugs. If a current smoker reported smoking in the last 30 days, it is defined as smoking.

Baseline data were collected at the time of enrollment. Peripheral venous blood samples were taken after overnight fasting for at least 8 h. Based on protocol, the blood samples were collected in the vacuum tube by the EDTA-anticoagulated plastic tubes and centrifuged within 1 h after collection. All the blood samples were centrifuged at 1,000 *g* for 10 min and then the serum was shipped within 2–4 h on dry ice to the laboratory and stored at −80°C. The blood glucose level was measured using commercial reagents following standard procedures. High-performance liquid chromatography (MQ-2000PT, Medconn, China) was used to assess the level of glycated hemoglobin (HbA1c). Beckman-coulter AU 680 was used to measure fasting plasma glucose, serum creatinine, triglyceride (TG), total cholesterol (TC), high-density lipoprotein cholesterol (HDL-C), and low-density lipoprotein cholesterol (LDL-C).

### Outcome Assessment

Patients were followed up until December 2020 or until the occurrence of cardiovascular death. All participants were followed up by analyses of clinical materials and telephone contact quarterly. The endpoint was cardiovascular death. Cardiovascular death was defined as deaths caused by coronary heart disease or stroke and deaths that cannot be classified without evidence of the source. Unless a clear non-cardiac cause is established, all deaths are considered cardiac deaths based on the International Classification of Diseases codes for confirmation of each cause of death or routine death registration. Excluding the participants who were lost to follow-up, we obtained follow-up of all patients until the primary outcome or date of censoring. The follow-up time was calculated from the date of cardiac death onset to the date of mortality occurrence or the date of the last follow-up. Written informed content was obtained from all study participants, and the study was approved by the ethics committee of Chinses PLA General Hospital.

### Statistical Analysis

Patients were first divided into three groups according to the levels of WC. The cut-off point for the WC was 82.94. Then, the patients were further categorized into six groups as WC with DM and non-DM. Variables with a normal distribution are expressed as the mean ± SD, and in the case of non-normality, the medians are presented. Categorical data are expressed in counts or percentages. Chi-square tests and ANOVA were used to assess baseline differences between the three groups. The Kaplan–Meier method was used to evaluate the cumulative risk of the outcome at different WC levels with or without DM and compared by log-rank tests. Univariate and multivariable-adjusted Cox regression models were used to assess the association of WC and diabetes with the endpoints. The results are presented as the hazard ratios (HRs) and 95% CIs according to levels of WC. Four multivariate proportional hazards models were fitted. Model 1 contains the following variables: age, sex, BMI, current smokers, hypertension, TC, TG, HDL-C, and LDL-C. Variables were input into the model according to their statistical significance in univariate analysis. Model 2 was based on model 1 with the addition of WC. Model 3 contains model 1 with the addition of diabetes. Model 4 contains model 1 with the addition of WC and diabetes. Receiver operating characteristic curve analysis was used to compare the prognostic powers of the 4 models. In addition, when WC or diabetes was added to the established model, continuous net weight classification index (NRI) and comprehensive discrimination improvement (IDI) were generated to assess any improvement in prognostic prediction. SPSS 22.0 and R 4.0.0 (R Foundation for Statistical Computing) were used for descriptive data analysis. All statistical tests were 2-tailed, and *p*-values <0.05 were considered statistically significant.

## Results

### Baseline Characteristics

Baseline measurements of WC were available in 1,521 patients. We divided the patients into three groups based on the levels of WC. The baseline clinical and laboratory characteristics of the study patients are presented in Table 1. The patients with a higher WC level were older, more likely to be female, more likely to have diabetes. Moreover, they had a higher BMI, hips circumference, fasting blood glucose, 2-h postprandial blood glucose (2hPBG) level, HbA1c level, and uric acid level. Also, they had higher systolic blood pressure (SBP) and diastolic blood pressure (DBP) levels. However, they had lower HDL-C and TC levels.

**Table 1:** Baseline characteristics of the subjects by the waist circumference level.

**Variables**		**Waist circumference level**			
	**Total**	** <82 cm**	**82–94 cm**	**>94 cm**	***p*-value for trend**
	**(*n* = 1521)**	**(*n* = 438)**	**(*n* = 752)**	**(*n* = 331)**	
Age, years	70.96 ± 7.18	69.98 ± 7.23	70.86 ± 6.90	72.49 ± 7.48	0.000
Male, *n* (%)	900 (59.4)	336 (76.71)	430 (57.18)	134 (40.48)	0.000
T2DM, *n* (%)	449 (29.52)	106 (24.20)	215 (28.59)	128 (38.67)	0.000
Hypertension, *n* (%)	785 (51.61)	174 (39.73)	410 (54.52)	201 (60.73)	0.411
Current smokers, *n* (%)	253 (16.63)	84 (19.18)	113 (15.03)	56 (16.92)	0.531
BMI (kg/m^2^)	24.88 ± 3.46	21.98 ± 2.84	25.01 ± 2.31	28.39 ± 2.98	0.000
Hips circumference (cm)	97.97 ± 8.36	90.88 ± 7.40	98.54 ± 5.24	106.06 ± 7.35	0.000
Fasting blood glucose (mmol/L)	5.68 ± 0.99	5.46 ± 0.77	5.67 ± 1.02	5.97 ± 1.12	0.000
2hPBG (mmol/L)	8.08 ± 3.29	7.19 ± 2.73	8.19 ± 3.32	9.00 ± 3.61	0.000
HbA1c	5.86 ± 1.045	5.70 ± 0.62	5.87 ± 1.17	6.04 ± 1.17	0.000
SBP (mmHg)	137.42 ± 19.13	132.77 ± 17.30	138.43 ± 17.89	141.36 ± 19.51	0.000
DBP (mmHg)	76.97 ± 9.96	73.84 ± 8.74	77.79 ± 9.28	79.23 ± 9.21	0.000
TC (mmol/L)	5.29 ± 0.99	5.37 ± 0.97	5.29 ± 1.01	5.16 ± 0.95	0.016
HDL-C (mmol/L)	1.44 ± 0.39	1.62 ± 0.42	1.40 ± 0.34	1.30 ± 0.39	0.000
LDL-C (mmol/L)	3.26 ± 0.85	3.24 ± 0.84	3.30 ± 0.92	3.17 ± 0.85	0.055
TG (mmol/L)	1.63 ± 0.82	1.42 ± 0.76	1.65 ± 0.82	1.85 ± 0.82	0.341
Creatinine (umol/L)	74.46 ± 22.22	70.64 ± 24.98	74.42 ± 19.77	79.62 ± 22.64	0.271
Uric acid (umol/L)	309.06 ± 89.27	282.59 ± 74.79	307.99 ± 91.22	346.61 ± 89.54	0.000

### Association Between WC and Diabetes and Prognosis of Cardiovascular Death

**Figure 2:**
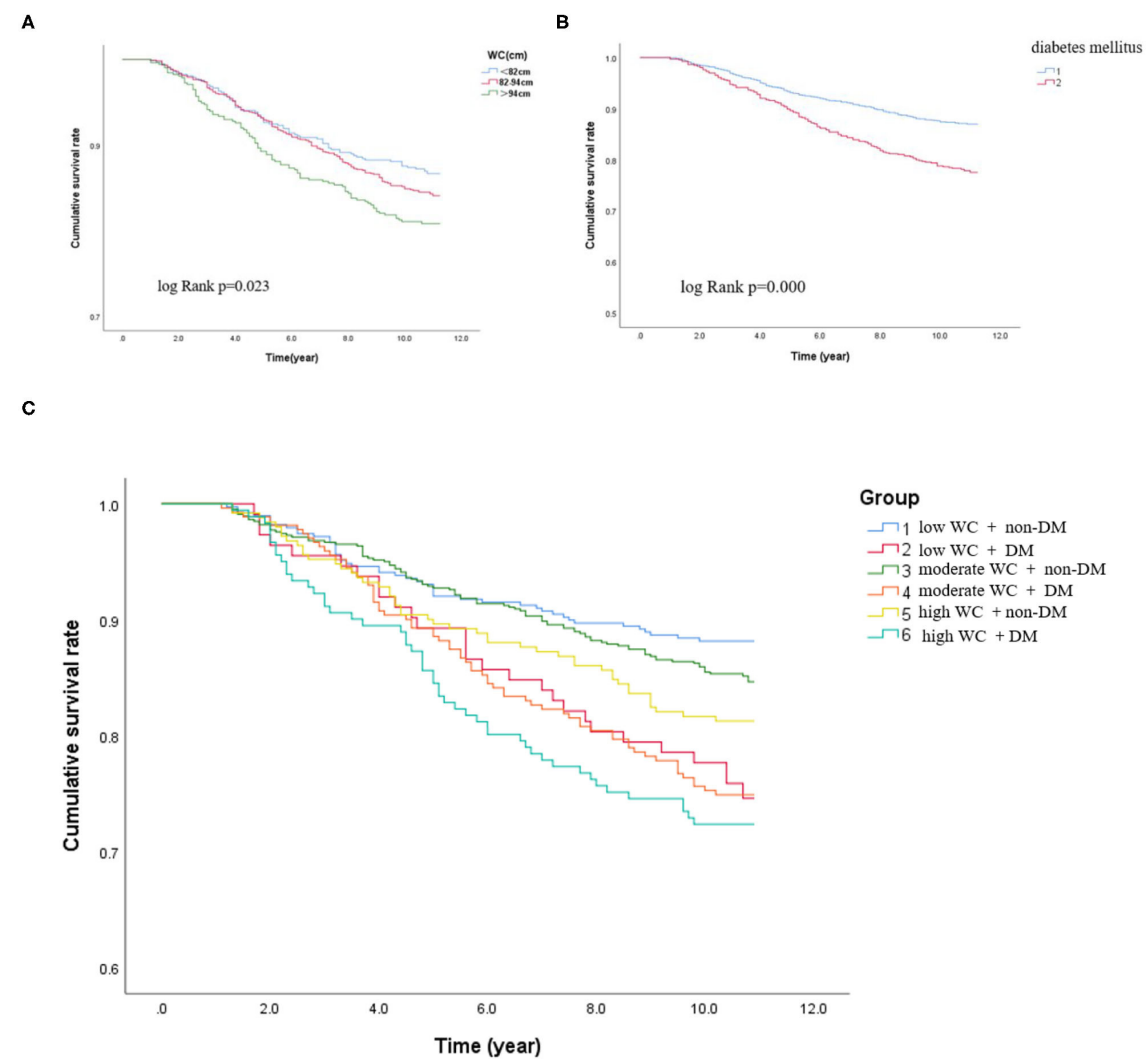
Kaplan–Meier curves in different subgroups. **(A)** Kaplan–Meier survival rate of the participants according to the different WC levels. **(B)** Kaplan–Meier survival rate of the participants with or without DM. **(C)** Kaplan–Meier survival rate of the participants with DM and non-DM according to different degrees of WC.

**Figure 3:**
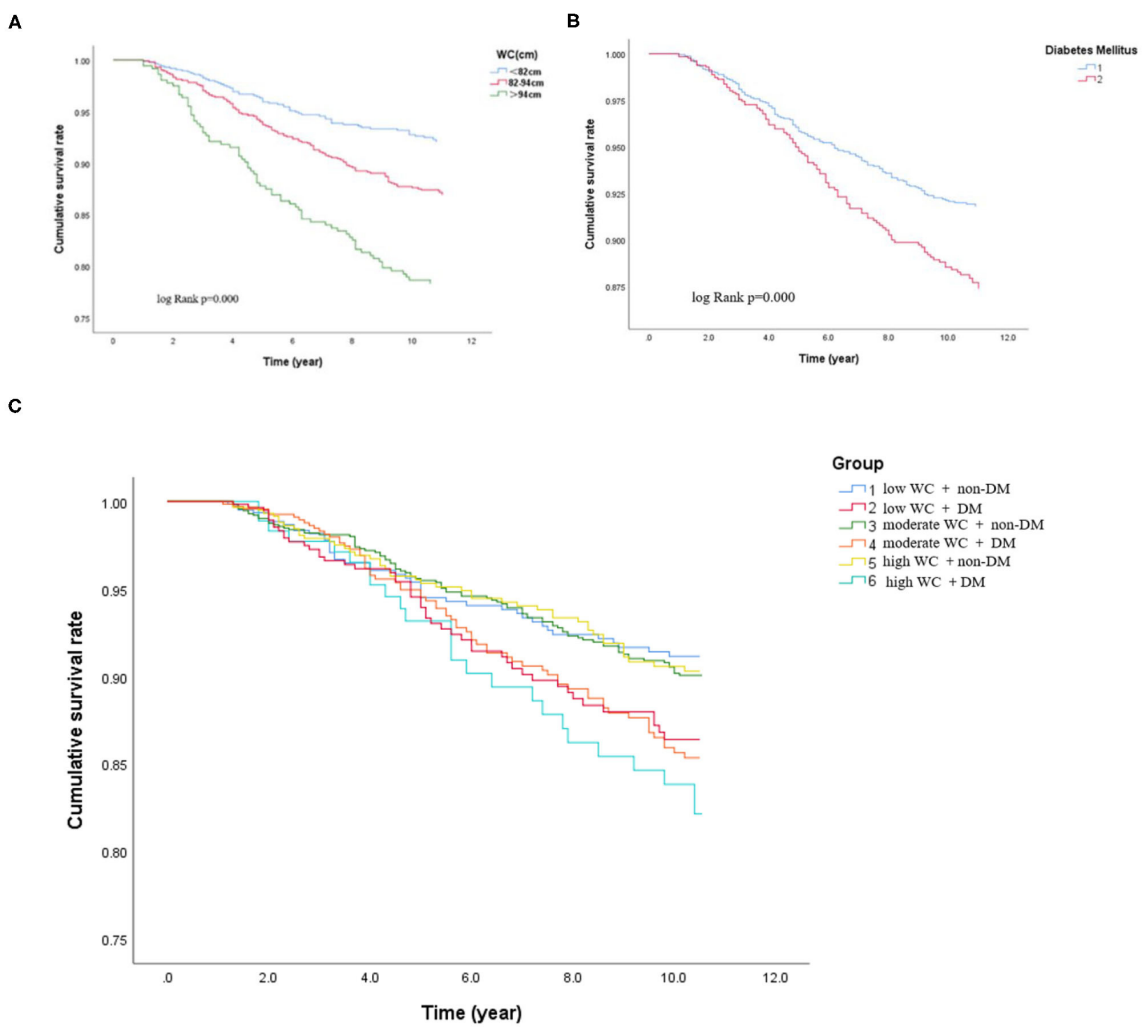
Cox-adjusted event-free survival curves for freedom of cardiovascular death in the different subgroups after adjustment for potential clinical confounders. **(A)** Cox-adjusted event-free survival curves of the participants according to the different WC levels. **(B)** Cox-adjusted event-free survival curves of the participants with or without DM. **(C)** Cox-adjusted event-free survival curves of the participants with DM and non-DM according to different degree of WC.

During the median follow-up of 9.2 years, 265 participants had the occurrence of cardiovascular death. The death rate of the higher WC group was significantly higher than the lower group (23.3 vs. 16.0 vs. 15.0%, *p* < 0.05). Kaplan–Meier curves were used to show the cumulative event curves for cardiovascular death stratified according to WC levels (Figure 2A) and diabetes (Figure 2B): patients with a higher level of WC or patients with diabetes were more likely to have a higher cardiovascular death rate (log-rank test, *p* < 0.05, respectively). Table 2 shows the univariate and multivariate Cox regression analyses of cardiovascular death predictors. After adjusting for potential confounding factors, patients with higher WC level group (WC > 94 cm) (adjusted HR: 3.02; 95% CI: 1.88–3.83; *p* = 0.001) (Figure 3A) or diabetes (adjusted HR: 1.59; 95% CI: 1.46–1.77; *p* < 0.001) (Figure 3B) had a significantly higher risk of cardiovascular death. Age, BMI, hypertension, hips circumference, fasting blood glucose, 2hPBG, HbA1c, SBP, DBP, current smoking, hypertension, DM, TC, TG, HDL-C, LDL-C, creatinine, and uric acid were included in the confounding factors.

**Table 2:** Univariate and multivariate Cox regression analyses of cardiovascular death predictors.

**Variables**	**Univariable analysis**			**Multivariable analysis**		
	**Crude HR**	**95%CI**	***P*-value**	**Crude HR**	**95%CI**	***p*-value**
Age, years	1.18	1.16–1.20	0.00	1.16	1.13–1.18	0.00
Male, *n* (%)	1.02	0.96–1.03	0.12	–	–	
BMI (kg/m^2^)	0.97	0.96–0.98	0.00	0.95	0.90–1.01	0.09
Hips circumference (cm)	0.99	0.97–1.00	0.07	–	–	
Fasting blood glucose (mmol/L)	1.08	1.03–1.14	0.00	1.09	0.92–1.30	0.34
2hPBG (mmol/L)	1.03	0.99–1.06	0.14	–	–	
HbA1c	1.11	1.04–1.19	0.00	1.04	0.92–1.19	0.53
SBP (mmHg)	1.01	1.002–1.012	0.01	1.00	0.99–1.01	0.25
DBP (mmHg)	0.99	0.98–0.99	0.03	0.99	0.97–1.00	0.11
T2DM, *n* (%)	1.56	1.45–1.69	0.00	1.59	1.46–1.77	0.00
Hypertension, *n* (%)	1.07	0.89–1.26	0.00	1.08	0.85–1.22	0.06
Current smokers, *n* (%)	1.02	0.89–1.07	0.18	–	–	
TC (mmol/L)	0.75	0.68–0.85	0.00	1.28	0.64–2.57	0.49
HDL-C (mmol/L)	0.97	0.74–1.28	0.84	–	–	
LDL-C (mmol/L)	0.78	0.69–0.88	0.00	0.75	0.36–1.55	0.44
TG (mmol/L)	0.71	0.61–0.83	0.00	0.58	0.42–0.80	0.00
Creatinine (umol/L)	1.01	1.008–1.013	0.00	1.01	1.01–1.02	0.00
Uric acid (umol/L)	1.003	1.002–1.004	0.00	1.002	1.001–1.004	0.00
Group
WC <82 cm	1	–	–	1	–	–
WC: 82–94 cm	1.06	0.53–1.32	0.05	1.68	1.18–2.41	0.01
WC > 94 cm	1.59	0.97–2.57	0.07	3.02	1.88–3.83	0.00

In subgroups, the patients were further categorized into six groups high WC with DM and non-DM, moderate WC with DM and non-DM, and low WC with DM and non-DM. The incidence of cardiovascular death was significantly higher when patients with high WC coexisted with DM (low WC with DM or non-DM: 25.9 vs. 11.8%; moderate WC with DM or non-DM: 26.9 vs. 14.9%; high WC with DM or non-DM: 27.6 vs. 18.7%, *p* < 0.05, respectively). Patients with higher level of WC (WC > 94 cm) with diabetes have the highest cardiovascular death rate (27.6%) (log-rank test, *p* < 0.05). For patients with a low WC and non-DM, the cardiovascular death rate was 11.8%. Therefore, the risk of cardiovascular death in patients with high WC and diabetes was 2.3 times higher than that in patients with low WC and diabetes. Besides, as shown in Figure 2C, the Kaplan–Meier analysis curves presented the highest risk in patients with high WC with DM compared with other subgroups (log-rank test, *p* < 0.001). Multivariate Cox regression analysis showed that patients with a higher level of WC were more likely to have a higher cardiovascular death rate (adjusted HR = 3.78; 95% CI: 3.35–3.98; *p* < 0.001) (Figure 3C).

### Construction of the Prediction Models and the Model Performance

We adjusted four prediction models to evaluate the prognostic value. Model 1 was adjusted by the clinical confounders (age, sex, BMI, hips circumference, fasting blood glucose, 2hPBG, HbA1c, SBP, DBP, current smokers, hypertension, TC, HDL-C, LDL-C, TG, creatinine, and uric acid). Model 2 included model 1 with the addition of WC, model 3 included model 1 with the addition of WC, and model 4 included model 1 with the addition of WC + DM.

Finally, to evaluate the effect of WC and diabetes on the accuracy of cardiovascular death risk assessment, discriminatory abilities were compared between models with WC and diabetes. As shown in Table 3, adding WC and diabetes can significantly increase the static value of C from 0.693 (95%CI: 0.656–0.730) to 0.847 (95%CI: 0.724–0.891). Compared with the clinical model, there is a significant difference (*p* < 0.01). In addition, adding WC category to model 1 can significantly improve NRI = 0.195 (95% CI: 0.135–0.203, *p* < 0.05); IDI = 0.009 (95% CI: 0.003–0.014, *p* < 0.001) (Table 3).

**Table 3:** Reclassification and discrimination statistics for clinical outcomes by WC level and T2DM.

	**Model**	**Estimate (95%CI)**	***p*-value**
C static	Model 1	0.693 (95%CI: 0.656–0.730)	–
	Model 2: Model 1 + WC	0.812 (95%CI: 0.782–0.842)	0.031
	Model 3: Model 1 + DM	0.785 (95%CI: 0.769–0.812)	0.025
	Model 4: Model 1 + WC + DM	0.847 (95%CI: 0.724–0.891)	0.001
NRI	Model 1	REF	–
	Model 2: Model 1 + WC	0.178 (95% CI: 0.094–0.262)	0.042
	Model 3: Model 1 + DM	0.182 (95% CI: 0.135–0.203)	0.031
	Model 4: Model 1 + WC + DM	0.195 (95% CI: 0.135–0.203)	0.012
IDI	Model 1	REF	–
	Model 2: Model 1 + WC	0.012 (95% CI: 0.007–0.015)	0.229
	Model 3: Model 1 + DM	0.014 (95% CI: 0.013–0.020)	0.174
	Model 4: Model 1 + WC + DM	0.009 (95% CI: 0.006–0.014)	0.030

## Discussion

### Principal Findings

**Figure 4:**
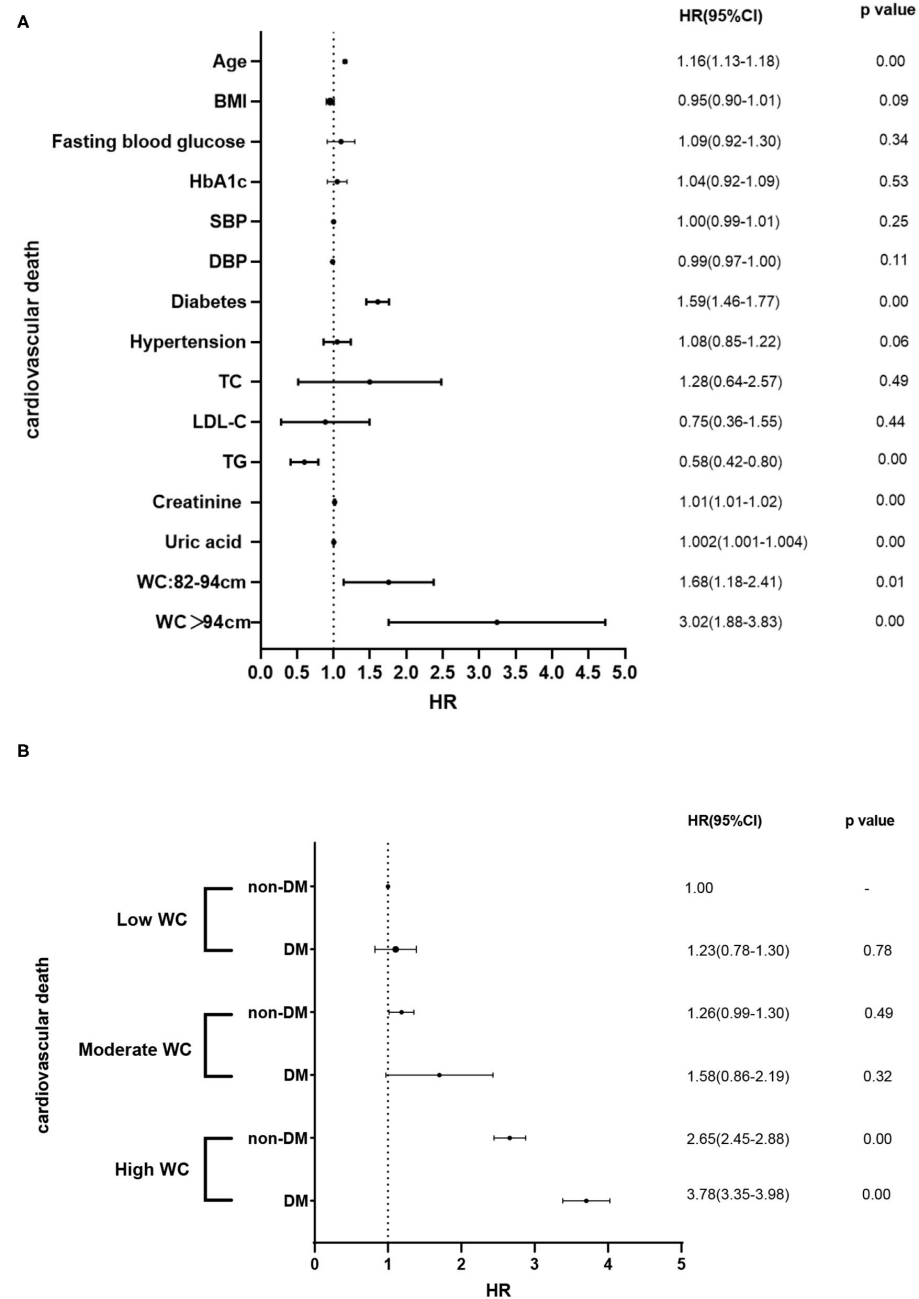
**(A)** Associations of clinical confounders and the prevalence of cardiovascular death. **(B)** Associations of different degrees of WC and DM and the prevalence of cardiovascular death in different subgroups.

In this perspective, observational study on a large Chinese cohort with long-term follow-up, we examined the prognostic association of DM with outcomes in patients with different degrees of WC. Our results suggested that higher WC and diabetes were both significant and independent predictors of cardiovascular death (Figure 4A). Patients with a higher level of WC (WC > 94 cm) with diabetes have the highest cardiovascular death rate (27.6%) (log-rank test, *p* < 0.05). For patients with a low WC and non-DM, the cardiovascular death rate was 11.8%. Therefore, the risk of cardiovascular death in patients with high WC and diabetes was 2.3 times higher than that in patients with low WC and diabetes. In addition, our results suggested that patients with higher levels of WC coexisted with diabetes had the worst outcome, the association is still significant after adjustment of other clinical confounders (Figure 4B). In summary, our results suggested that the addition of WC and diabetes to established cardiovascular risk factors may further improve risk stratification in the general population. Our results provided updated information about the long-term prognostic role of WC and diabetes in the general population.

### Limitations of BMI

Adipose tissue is now considered to be a key organ for the fates of excessive dietary lipids, which may determine whether it will maintain body homeostasis (metabolic healthy obesity) or whether it will produce inflammation/insulin resistance, which can have harmful cardiovascular consequences. Obesity, especially visceral obesity, can also cause various structural adaptations/changes in the structure/function of CV. Adipose tissue can now be regarded as an endocrine organ that coordinates important interactions with vital organs and tissues (such as the brain, liver, skeletal muscle, heart, and blood vessels themselves).

The most commonly used anthropometric tool for assessing relative weight and classifying obesity is BMI, which is expressed as the ratio of total body weight to the square of height (kg/m^2^). Individuals with a BMI <18.5 kg/m^2^ are considered underweight, while individuals with a BMI between 18.5 and 24.9 kg/m^2^ are classified as normal or acceptable weight. Individuals with a BMI between 25 and 29.9 kg/m^2^ are classified as overweight, while those with a BMI of ≥30 kg/m^2^ are obese. BMI itself is associated with clinical outcomes and mortality in a U or J type (10). This inverse relationship has triggered controversy in the literature, called the “obesity paradox” (11). Compared with non-obese patients, patients with elevated BMI with chronic diseases have a higher survival rate and fewer cardiovascular (CV) events (12). Moreover, previous research reported that patients with an increased BMI were found to show lower mortality (13). In addition, BMI cannot distinguish between weight gain due to high levels of lean body mass and fat body mass. Generally, excess body fat (BF) is more often associated with metabolic abnormalities than high levels of lean body mass. Another explanation for this paradox is the lack of control over the main individual differences in the regional BF distribution. Therefore, more and more scholars believe that BMI has its limitations to fully capture cardiometabolic risk. It is partially related to the fact that BMI in isolation is an insufficient biomarker of abdominal adiposity (4). By using the BMI, one must rely on the assumption that adipose tissue is distributed evenly over the body, which does not take into account the heterogeneity of regional body fat deposition (14).

The level of obesity must be considered in the risk stratification. In a recent meta-analysis of 2.88 million people, all levels of obesity combined were associated with increased mortality, with an HR of 1.18 (95% CI: 1.12–1.25). However, when analyzing separately, compared with normal body weight, grade 1 obesity (Table 1) is associated with a risk of death with an HR of 0.97 (95% CI, 0.90–1.04), in contrast, serious obesity (grades 2 and 3) had a higher risk of death (HR: 1.34, 95% CI: 1.21–1.47) (15). The prognostic value of BMI needs to pay attention to the length of follow-up time. There was a J-type association between BMI and sudden cardiac death, and the lowest risk was observed within the normal weight range. However, in studies with a longer follow-up period, the increased risk of low BMI was attenuated (16). In other words, the obesity phenotype may change over time to reflect the increase in abdominal obesity. For example, Ian Janssen et al. studied the changes in WC for a given BMI over 30 years in a Canadian sample 35. It is worth noting that for a given BMI, Canadians had a larger WC in 2007 than in 1981. Specifically, the researchers observed that between 1981 and 2007 men with a BMI of 25 kg/m^2^ increased their WC by 1.1 cm, and women with a BMI of 25 kg/m^2^ increased their WC by 4.9 cm. Similarly, Sandra Albrecht et al. studied 36 long-term changes in WC in the United States (1988–2007), the United Kingdom (1992–2008), China (1993–2011), and Mexico (1999–2012) and reported significant statistics academic significance in all countries and most subgroups, WC values have increased relative to BMI. The result of one study involving more than 58,000 elderly persons, during a 5-year-follow-up, showed increased mortality risks for elderly people with an increased WC—even across BMI categories—and for those who were classified as “underweight” using BMI. Part of the reason why BMI cannot fully capture cardiometabolic risks is that BMI alone is an insufficient biomarker for the whole body. More importantly, the central abdominal fat mass does not explain the extreme changes in intra-abdominal (visceral) fat mass and fat distribution between individuals (17). Compared with BMI, WC has a higher predictive value for cardiovascular death (18).

### Visceral Adipose Tissue and the Underlying Mechanisms

Visceral adipose tissue (VAT) has been proved to be independently associated with elevated CVD risk (19). Data from several past epidemiological studies 30 years of experience show that VAT is an independent sign of morbidity and mortality (20). In some populations, WC is more predictive of overall mortality, coronary heart disease (CHD). However, prospective data on the impact of abdominal obesity on CVD incidence is still scarce. Many experimental studies support the potential connection between VAT and biological pathways that are important in the pathogenesis of multiple disease outcomes. Adipokines are biologically active molecules secreted by adipose tissue and are key components of these pathways, including inflammatory cytokines, angiogenic factors, lipid metabolites, and extracellular matrix components (21). The secretion of adipokines among specific fat depots appears to be different (22), and compared with subcutaneous adipose tissue (SAT), VAT exhibits more pro-inflammatory and proangiogenic gene expression. In addition, compared with SAT, small arteries in VAT are more likely to exhibit endothelial dysfunction (23), indicating that VAT has a potentially toxic effect on the vasculature. Visceral adipocytes differ from subcutaneous adipocytes in that they release secreted proteins that are known or potential risk factors for CHD. In at least one study, visceral fat expressed and released more plasminogen activator inhibitor-1, a fibrinolysis inhibitor, than subcutaneous fat (24). Angiotensinogen is a potential blood pressure regulator and is also highly expressed in VAT.

There are currently multiple methods to assess body fat distribution. The most accurate method is costly and time-consuming. It is not suitable for large-scale population research. Since routine access to CT, MRI may be too expensive to be feasible for many clinicians, and the use of these methods to image visceral and ectopic fat has historically been reserved for research purposes, perhaps the most widely used, and these measurements are taken for WC. Ashwell et al. were the first to show that there is a correlation between visceral fat mass and waist-to-hip ratio. However, compared with the waist-to-hip ratio, WC has a stronger correlation with visceral fat mass (25).

### Obesities, Type 2 Diabetes Mellitus, and Cardiovascular Outcomes

Obesity is an important driving factor for the development of type 2 diabetes. Compared with BMI-matched non-type 2 diabetic patients, type 2 diabetic patients have a larger WC and more VAT (7). A patient with VAT or severe obesity and type 2 diabetes is susceptible to cardiovascular abnormalities and CVD; their simultaneous presence should further increase the risk of cardiovascular outcomes (26). The underlying mechanisms may be ectopic, and visceral obesity is related to insulin resistance, this may partially mediate the link between obesity, type 2 diabetes, and cardiovascular risk. Metabolic syndrome and insulin resistance have been recognized as risk factors for cardiovascular morbidity and mortality (27). Studies have also shown that the presence of metabolic syndrome increases the risk of heart failure. The result of a cross-sectional study has shown that obesity and type 2 diabetes have an additive effect on left ventricular remodeling in normotensive patients (28). A recent study of patients with type-2 DM showed that compared with those in the first quartile of WC, male patients in the fourth quartile of WC (WC ≥ 126) had an HR of 1.24 (95% CI: 1.05–1.46) for major adverse cardiovascular events (MACEs); female patients in the fourth quartile of WC (WC ≥ 122 cm) had an HR of 1.22 (95% CI: 0.96–1.56) for MACEs (29). Our results suggested that patients with higher levels of WC coexisted with diabetes had the worst outcome, the association is still significant after adjustment of other clinical confounders. Our results provided updated information about the long-term prognostic role of WC and diabetes in the general population.

## Limitations of Our Study

Our study still has some limitations: first, we calculate the WC at bassline, we did not reevaluate WC during the follow-up. However, previous studies showed that changes in WC were not significantly associated with mortality (30). Second, all included patients were Asians, and the result may not apply to another population. Another limitation would be the lack of “gold standard” methods for abdominal obesity, such as CT or MRI.

## Conclusion

In conclusion, our study demonstrated that an increased WC (WC ≥ 94 cm) was associated with increased cardiovascular death in the general population. Patients with higher levels of WC coexisted with diabetes had the worst outcome. WC and diabetes may provide incremental prognostic value beyond traditional risks factors.

## Data Availability Statement

The raw data supporting the conclusions of this article will be made available by the authors, without undue reservation.

## Ethics Statement

The studies involving human participants were reviewed and approved by Chinese PLA General Hospital. The patients/participants provided their written informed consent to participate in this study.

## Author Contributions

S-xW and PZ made substantial contributions to the conception or design of the work. ML contributed to the data collection, data interpretation, and critical review and drafting of the manuscript. All authors read and approved the final manuscript.

## Funding

This work was supported by the National Key Research and Development Program of China (2020YFC2008900), the National Defense Science and Technology Innovation Special Zone Project (19-163-15-ZD-009-001-10), and the Key Projects of Logistics Scientific Research Project of Chinese PLA (19BJZ30).

## Conflict of Interest

The authors declare that the research was conducted in the absence of any commercial or financial relationships that could be construed as a potential conflict of interest.

## Publisher's Note

All claims expressed in this article are solely those of the authors and do not necessarily represent those of their affiliated organizations, or those of the publisher, the editors and the reviewers. Any product that may be evaluated in this article, or claim that may be made by its manufacturer, is not guaranteed or endorsed by the publisher.

## Abbreviations

AUC: area under the curve
BF: body fat
BMI: body mass index
CV: cardiovascular
CVD: cardiovascular disease
CHD: coronary heart disease
DBP: diastolic blood pressure
T2DM: type 2 diabetes mellitus
HDL-C: high-density lipoprotein cholesterol
HR: hazard ratio
IDI: integrated discrimination improvement
LDL-C: low-density lipoprotein cholesterol
MACEs: major adverse cardiovascular events
NRI: net reclassification index
SAT: subcutaneous adipose tissue
SBP: systolic blood pressure
SD: standard deviation
TC: total cholesterol
TG: triglyceride
VAT: visceral adipose tissue
WC: waist circumference.

## References

[1] Piché M-E Tchernof A Després J-P. Obesity phenotypes, diabetes, and cardiovascular diseases. Circ Res. (2020) 126:1477–500. 10.1161/CIRCRESAHA.120.316101 32437302

[2] Kim R Kawachi I Coull BA Subramanian SV. Contribution of socioeconomic factors to the variation in body-mass index in 58 low-income and middle-income countries: an econometric analysis of multilevel data. Lancet Glob Health. (2018) 6:e777–86. 10.1016/S2214-109X(18)30232-8 29903378

[3] Neeland IJ Ross R Després J-P Matsuzawa Y Yamashita S Shai I. Visceral and ectopic fat, atherosclerosis, and cardiometabolic disease: a position statement. Lancet Diabetes Endocrinol. (2019) 7:715–25. 10.1016/S2213-8587(19)30084-1 31301983

[4] Ross R Neeland IJ Yamashita S Shai I Seidell J Magni P. Waist circumference as a vital sign in clinical practice: a Consensus Statement from the IAS and ICCR Working Group on Visceral Obesity. Nat Rev Endocrinol. (2020) 16:177–89. 10.1038/s41574-019-0310-7 32020062 PMC7027970

[5] Fox CS Massaro JM Hoffmann U Pou KM Maurovich-Horvat P Liu C-Y. Abdominal visceral and subcutaneous adipose tissue compartments: association with metabolic risk factors in the Framingham Heart Study. Circulation. (2007) 116:39–48. 10.1161/CIRCULATIONAHA.106.675355 17576866

[6] Kaess BM Pedley A Massaro JM Murabito J Hoffmann U Fox CS. The ratio of visceral to subcutaneous fat, a metric of body fat distribution, is a unique correlate of cardiometabolic risk. Diabetologia. (2012) 55:2622–30. 10.1007/s00125-012-2639-5 22898763 PMC3636065

[7] Balkau B Deanfield JE Després J-P Bassand J-P Fox KAA Smith SC. International Day for the Evaluation of Abdominal Obesity (IDEA): a study of waist circumference, cardiovascular disease, and diabetes mellitus in 168,000 primary care patients in 63 countries. Circulation. (2007) 116:1942–51. 10.1161/CIRCULATIONAHA.106.676379 17965405 PMC2475527

[8] Gallagher D Kelley DE Yim J-E Spence N Albu J Boxt L. Adipose tissue distribution is different in type 2 diabetes. Am J Clin Nutr. (2009) 89:807–14. 10.3945/ajcn.2008.26955 19158213 PMC2714397

[9] Karlsson T Rask-Andersen M Pan G Höglund J Wadelius C Ek WE. Contribution of genetics to visceral adiposity and its relation to cardiovascular and metabolic disease. Nat Med. (2019) 25:1390–5. 10.1038/s41591-019-0563-7 31501611

[10] van der Meer TGLA Verhoeven P Beentjes JWJ Vliegenthart R. Disrupting gatekeeping practices: journalists' source selection in times of crisis. Journalism. (2017) 18:1107–24. 10.1177/1464884916648095 29278263 PMC5732591

[11] Elagizi A Kachur S Lavie CJ Carbone S Pandey A Ortega FB. An overview and update on obesity and the obesity paradox in cardiovascular diseases. Prog Cardiovasc Dis. (2018) 61:142–50. 10.1016/j.pcad.2018.07.003 29981771

[12] Bastien M Poirier P Lemieux I Després J-P. Overview of epidemiology and contribution of obesity to cardiovascular disease. Prog Cardiovasc Dis. (2014) 56:369–81. 10.1016/j.pcad.2013.10.016 24438728

[13] Morse SA Gulati R Reisin E. The obesity paradox and cardiovascular disease. Curr Hypertens Rep. (2010) 12:120–6. 10.1007/s11906-010-0099-1 20424935

[14] Després JP Lemieux I Prud'homme D. Treatment of obesity: need to focus on high risk abdominally obese patients. BMJ. (2001) 322:716–20. 10.1136/bmj.322.7288.716 11264213 PMC1119905

[15] Flegal KM Kit BK Orpana H Graubard BI. Association of all-cause mortality with overweight and obesity using standard body mass index categories: a systematic review and meta-analysis. JAMA. (2013) 309:71–82. 10.1001/jama.2012.113905 23280227 PMC4855514

[16] Aune D Schlesinger S Norat T Riboli E. Body mass index, abdominal fatness, and the risk of sudden cardiac death: a systematic review and dose-response meta-analysis of prospective studies. Eur J Epidemiol. (2018) 33:711–22. 10.1007/s10654-017-0353-9 29417316 PMC6061127

[17] Neeland IJ Poirier P Després J-P. Cardiovascular and metabolic heterogeneity of obesity: clinical challenges and implications for management. Circulation. (2018) 137:1391–406. 10.1161/CIRCULATIONAHA.117.029617 29581366 PMC5875734

[18] Bigaard J Frederiksen K Tjønneland A Thomsen BL Overvad K Heitmann BL. Waist circumference and body composition in relation to all-cause mortality in middle-aged men and women. Int J Obes. (2005) 29:778–84. 10.1038/sj.ijo.0802976 15917857

[19] Kouli GM Panagiotakos DB Kyrou I Georgousopoulou EN Chrysohoou C Tsigos C. Visceral adiposity index and 10-year cardiovascular disease incidence: the ATTICA study. Nutr Metab Cardiovasc Dis. (2017) 27:881–9. 10.1016/j.numecd.2017.06.015 28851556

[20] Hiuge-Shimizu A Kishida K Funahashi T Ishizaka Y Oka R Okada M. Absolute value of visceral fat area measured on computed tomography scans and obesity-related cardiovascular risk factors in large-scale Japanese general population (the VACATION-J study). Ann Med. (2012) 44:82–92. 10.3109/07853890.2010.526138 20964583

[21] Ouchi N Parker JL Lugus JJ Walsh K. Adipokines in inflammation and metabolic disease. Nat Rev Immunol. (2011) 11:85–97. 10.1038/nri2921 21252989 PMC3518031

[22] Hocking SL Wu LE Guilhaus M Chisholm DJ James DE. Intrinsic depot-specific differences in the secretome of adipose tissue, preadipocytes, and adipose tissue-derived microvascular endothelial cells. Diabetes. (2010) 59:3008–16. 10.2337/db10-0483 20841607 PMC2992760

[23] Farb MG Ganley-Leal L Mott M Liang Y Ercan B Widlansky ME. Arteriolar function in visceral adipose tissue is impaired in human obesity. Arterioscler Thromb Vasc Biol. (2012) 32:467–73. 10.1161/ATVBAHA.111.235846 22095978 PMC3262114

[24] Bruno MEC Mukherjee S Stromberg AJ Saito H Starr ME. Visceral fat-specific regulation of plasminogen activator inhibitor-1 in aged septic mice. J Cell Physiol. (2021) 237:706–19. 10.1002/jcp.30551 34369600 PMC8810697

[25] Snijder MB van Dam RM Visser M Seidell JC. What aspects of body fat are particularly hazardous and how do we measure them? Int J Epidemiol. (2006) 35:83–92. 10.1093/ije/dyi253 16339600

[26] Schramm TK Gislason GH Køber L Rasmussen S Rasmussen JN Abildstrøm SZ. Diabetes patients requiring glucose-lowering therapy and nondiabetics with a prior myocardial infarction carry the same cardiovascular risk: a population study of 3.3 million people. Circulation. (2008) 117:1945–54. 10.1161/CIRCULATIONAHA.107.720847 18378618

[27] Lakka H-M Laaksonen DE Lakka TA Niskanen LK Kumpusalo E Tuomilehto J. The metabolic syndrome and total and cardiovascular disease mortality in middle-aged men. JAMA. (2002) 288:2709–16. 10.1001/jama.288.21.2709 12460094

[28] De Jong KA Czeczor JK Sithara S McEwen K Lopaschuk GD Appelbe A. Obesity and type 2 diabetes have additive effects on left ventricular remodelling in normotensive patients-a cross sectional study. Cardiovasc Diabetol. (2017) 16:21. 10.1186/s12933-017-0504-z 28178970 PMC5299776

[29] Xing Z Peng Z Wang X Zhu Z Pei J Hu X. Waist circumference is associated with major adverse cardiovascular events in male but not female patients with type-2 diabetes mellitus. Cardiovasc Diabetol. (2020) 19:39. 10.1186/s12933-020-01007-6 32213183 PMC7093979

[30] Olson KL Neiberg RH Espeland MA Johnson KC Knowler WC Pi-Sunyer X. Waist circumference change during intensive lifestyle intervention and cardiovascular morbidity and mortality in the look AHEAD trial. Obesity. (2020) 28:1902–11. 10.1002/oby.22942 32881403 PMC7511417

